# TCM-DiffRAG: personalized syndrome differentiation reasoning method for traditional Chinese medicine based on knowledge graph and chain of thought

**DOI:** 10.3389/fmed.2026.1804478

**Published:** 2026-04-21

**Authors:** Jianmin Li, Ying Chang, Yujia Liu, Yanwen Wang, Binkai Ou, Shuyuan Lin, Su-Kit Tang, Lirong Zheng

**Affiliations:** 1 Faculty of Applied Sciences, Macao Polytechnic University, R. de Luís Gonzaga Gomes, Macao, Macao SAR, China; 2 Guangdong Institute of Intelligence Science and Technology, Zhuhai, Guangdong, China; 3 School of Basic Medical Sciences, Zhejiang Chinese Medical University, Hangzhou, China; 4 Zhejiang Chinese Medical University – GANCAO DOCTOR Institute of Artificial Intelligence for Chinese Medicine, Hangzhou, China; 5 Hangzhou Ganzhicao Technology Co., Ltd, Hangzhou, China; 6 BoardWare Information System Limited, Macao, Macao SAR, China

**Keywords:** chain-of-thought, knowledge graph, multi-step reasoning, retrieval-augmented generation, traditional Chinese medicine

## Abstract

**Background:**

Retrieval-augmented generation (RAG) technology can empower large language models (LLMs) to generate more accurate, professional, and timely responses without fine-tuning. However, due to the complex reasoning processes and substantial individual differences involved in traditional Chinese medicine (TCM) clinical diagnosis and treatment, traditional RAG methods often exhibit poor performance in this domain.

**Objective:**

To address the limitations of conventional RAG approaches in TCM applications, this study aims to develop an improved RAG framework tailored to the characteristics of TCM reasoning.

**Methods:**

We developed TCM-DiffRAG, an innovative RAG framework that integrates knowledge graphs (KG) with chains of thought (CoT). TCM-DiffRAG was evaluated on three distinctive TCM test datasets.

**Results:**

The experimental results demonstrated that TCM-DiffRAG achieved significant performance improvements over native LLMs. For example, the qwen-plus model achieved scores of 0.927, 0.361, and 0.038, which were significantly enhanced to 0.952, 0.788, and 0.356 with TCM-DiffRAG (McNemar test, *p* < 0.01). The improvements were even more pronounced for non-Chinese LLMs. Additionally, TCM-DiffRAG outperformed directly supervised fine-tuned (SFT) LLMs and other benchmark RAG methods.

**Conclusion:**

TCM-DiffRAG shows that integrating structured TCM knowledge graphs with Chain-of-Thought–based reasoning substantially improves performance in individualized diagnostic tasks. The joint use of universal and personalized knowledge graphs enables effective alignment between general knowledge and clinical reasoning. These results highlight the potential of reasoning-aware RAG frameworks for advancing LLM applications in traditional Chinese medicine.

## 1 Introduction

Since the end of 2022, large language models (LLMs) have made remarkable progress, and medical researchers have subsequently used LLMs in various clinical specialties. These preliminary studies (1–4) have shown that LLMs, with their powerful semantic understanding capabilities, exhibit significant advantages over traditional deep learning models in medical natural language processing tasks. However, while general-purpose LLMs have some effectiveness in the medical field, they still fall far short of expert levels (5), and sometimes they may produce incorrect or misleading content due to “hallucinations,” which may be related to the quality of training data, model architecture, training processes (6).

Although both fine-tuning (7–14) and RAG (15–25) have been widely practiced in the medical field, they still face many challenges in the field of TCM. Unlike modern medicine, the focus of TCM diagnosis is not on diseases, but on syndromes. A syndrome is a method of classifying pathological symptoms and signs to determine the body’s fundamental disorders. TCM obtains a patient’s syndrome through syndrome differentiation and treatment. TCM syndrome differentiation theory includes the Eight Principles, Zang-Fu organs, meridians, Qi and blood, or the Sanjiao theory (26). It analyzes the characteristics of syndromes and diseases based on different parameters such as the function of Zang-Fu organs, the combination of Yin and Yang meridians, and the circulation of Qi and blood (27). Although the principles of syndrome differentiation and treatment are the same, TCM can be divided into different schools, such as the Classical Formula school, the Earth school, and the Warm Disease school, during the specific diagnosis and treatment process, with certain differences among them (28). This situation is referred to as “treating different diseases with the same method” and “treating the same disease with different methods.”

Previous TCM large language models (14, 29) have done some work in continuous pre-training, supervised fine-tuning, and reinforcement learning. However, due to the lack of diagnostic and treatment data from different TCM schools, it is difficult to reflect the differences in clinical practice. At the same time, the RAG approach also faces numerous challenges in clinical scenarios. TCM clinical problems involve a large amount of potential reasoning. For problems with high complexity and strong logic, embedding models can only match text with surface similarity and cannot identify potential logical structures. In addition, the knowledge base in RAG often comes from textbooks, which is relatively theoretical and has a certain gap from real clinical practice. LLMs find it difficult to effectively derive coherent answers from fragmented or marginally relevant knowledge snippets (30).

To address these issues, we propose the TCM-DiffRAG method, with the following main contributions:

1. We propose a method for constructing a dual-level knowledge graph specifically for textbooks, further improving the quality of the RAG knowledge base.
2. By combining general knowledge graphs with actual cases, we construct a personalized knowledge graph, compensating for the lack of medical reasoning data and personalized diagnosis and treatment thinking in the knowledge base.
3. We train a TCM thinking chain model, which solves the problems of TCM school adaptation and the unity of retrieval comprehensiveness and focus in RAG practice.
4. We construct a set of evaluation datasets to verify the performance of the TCM thinking chain model and different knowledge bases.

## 2 Materials and methods

### 2.1 Related work

#### 2.1.1 Retrieval-augmented generation

**FIGURE 1:**
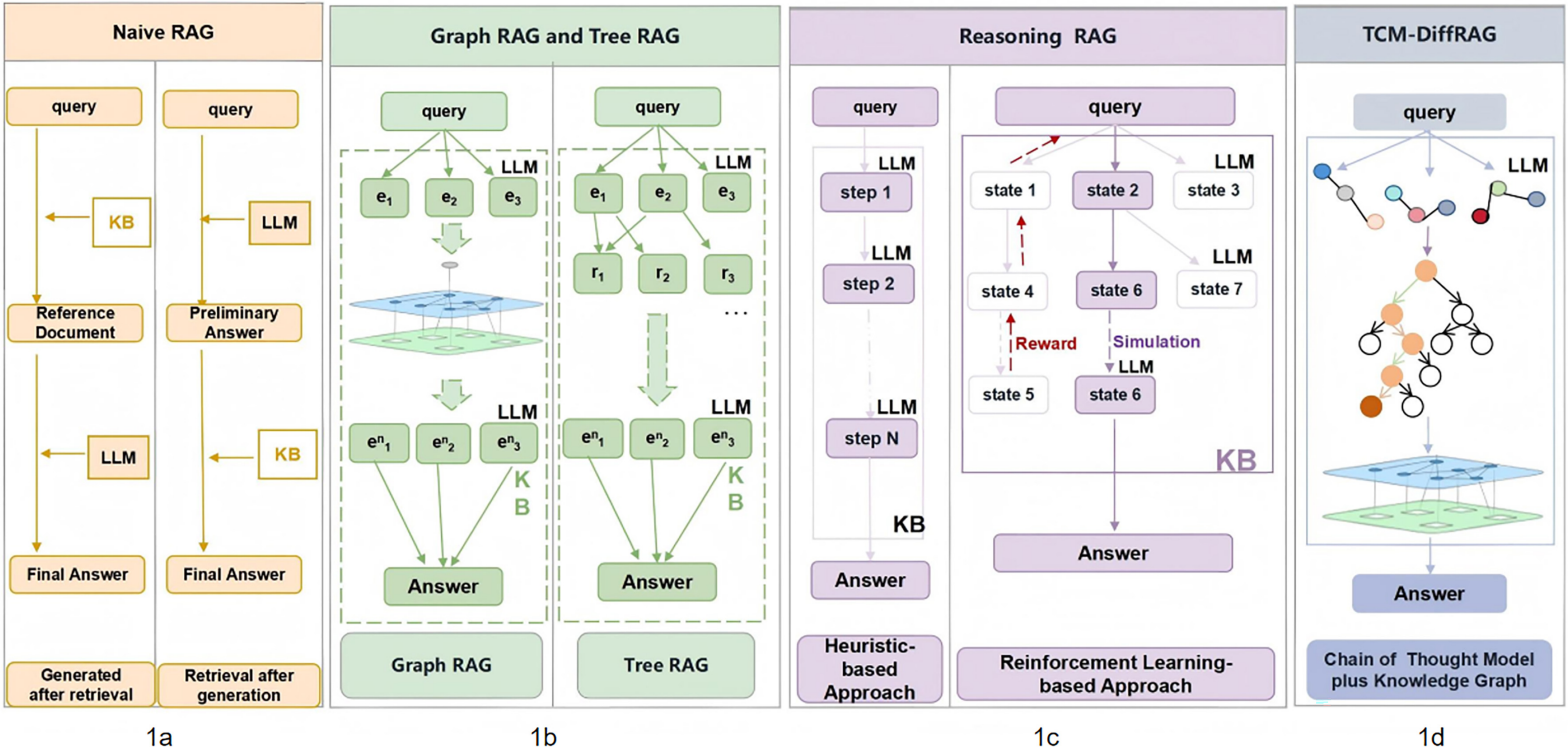
Comparison of several common RAG methods with the TCM-DiffRAG method. **(a)** Naive RAG, which generates answers after only one round of retrieval. Common approaches include generating answers after retrieval or retrieving after generating a preliminary answer. **(b)** RAG methods using different data structures as knowledge bases. Common approaches include those based on knowledge graphs and those based on thinking trees, which can improve the granularity of retrieval. **(c)** Reasoning-based RAG. Common approaches include heuristic methods and reinforcement learning-based methods. The former progressively decomposes complex questions based on prompt engineering, while the latter integrates the concepts of intelligent agents and reinforcement learning to train RAG systems capable of autonomously optimizing retrieval strategies and improving generation quality. **(d)** TCM-DiffRAG. Combining the characteristics of Knowledge Graph RAG and reasoning-based RAG, it uses a thinking chain model to decompose complex problems into triples, which are then retrieved and matched with the knowledge graph).

RAG technology requires additional model training and can dynamically integrate external knowledge bases to optimize model output, making it a research hotspot in medical LLM applications (31–33). The initial RAG, also known as Naive RAG, can be divided into three components: knowledge base, retriever, and large language model. Figure 1a shows the workflow of Naive RAG, where the user inputs a question, and the retriever (such as the BM25 algorithm or embedding model) retrieves documents from a static dataset. Then, the retrieved documents serve as context to enhance the generation capabilities of the large language model.

#### 2.1.2 Knowledge graph and RAG

Before the rise of LLMs, there were related studies on the combination of knowledge graphs and medical question-answering systems (34–38). After the rise of LLMs, knowledge graphs, due to their structured semantic associations, provide a new paradigm for deep reasoning of medical data and are often combined with RAG as a knowledge base component. As shown in Figure 1c, compared to Naive RAG, Knowledge Graph RAG can capture the relationships between entities in the question and recall documents with tighter semantic logic from the knowledge base. In addition, knowledge graphs can perform hierarchical knowledge management, improving the granularity of retrieval (39–42). Despite the practical effectiveness of Knowledge Graph RAG, it still faces significant challenges in constructing high-quality knowledge graphs (43) balancing the size of the retrieval subgraph with computational overhead (44), and the lack of multi-step reasoning (45).

#### 2.1.3 Reasoning RAG

Traditional question-answering systems are incapable of progressively decomposing complex questions and retrieving relevant information. As shown in Figure 1c, some studies have proposed reasoning-based QA systems, which employ methods such as prompt engineering to guide large language models in breaking down complex questions into multiple sub-questions for retrieval and reflection (46–48), or integrate intelligent agents and reinforcement learning concepts into reasoning-based retrieval (47, 49–51). Although reasoning-based QA systems can perform deep retrieval and reasoning, they fail to reflect personalized diagnostic thinking due to their reliance on generic knowledge bases and large models. To address this, we propose the TCM-DiffRAG method, as shown in Figure 1d. This approach effectively integrates knowledge graph-based RAG and reasoning-based RAG. It leverages knowledge graphs to ensure structured and high-quality knowledge, while utilizing reasoning-based retrieval to achieve precise and fine-grained information acquisition.

### 2.2. Methodology

#### 2.2.1 Construction of a general traditional Chinese medicine knowledge graph

**FIGURE 2:**
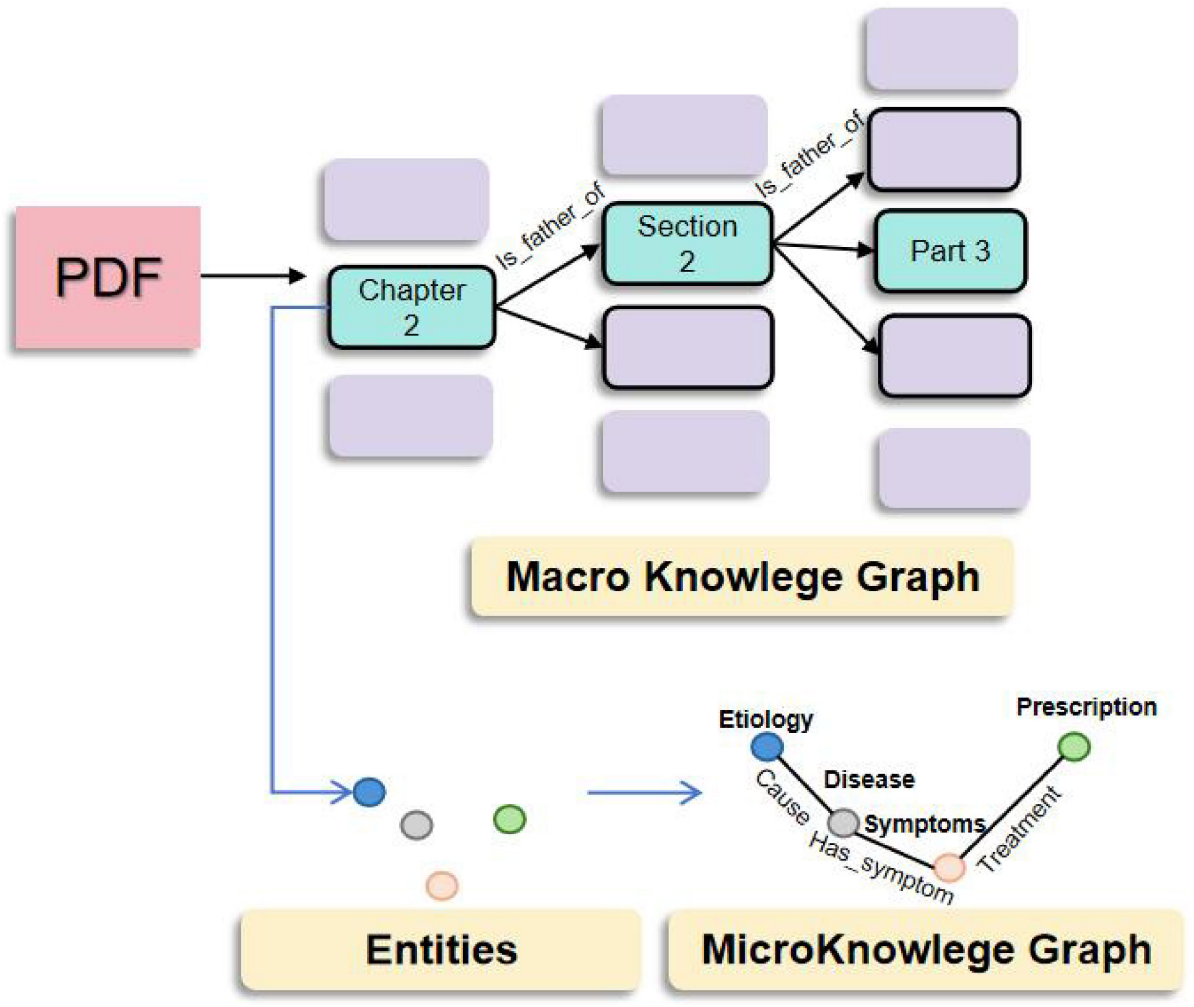
Schematic diagram of the general knowledge graph construction.

The quality of RAG is closely related to the quality of the knowledge base (17, 52). However, most previous studies on medical knowledge graphs and RAG (39, 41, 42) do not delve deeply into how to construct a knowledge graph. Therefore, we propose a “macro-micro” knowledge graph construction method specifically for medical textbooks. As shown in Figure 2, we collected 580 classic Chinese medicine textbooks, famous medical cases, and other books, and used previous document parsing research (53–56) for preprocessing. At the macro level of the books, we utilized a document layout model to identify the elements of each PDF page and extracted the titles and corresponding paragraph texts, constructing a knowledge graph similar to a tree diagram. The nodes consist of the titles of the books, and the node relationships are automatically generated through the parent-child structure of the titles. Although this structure sacrifices the traditional ontological concept of knowledge graphs, losing some rigor, it endows therapeutic knowledge with natural semantic indexing capabilities. At the micro level of medical entities, we extracted entities and relationships from paragraph texts using a large language model. The macro title nodes serve as structural hubs, establishing bidirectional mappings with micro entities.

Let the document set D be composed of a chapter hierarchy structure *H* and a content collection *P*:


D={ℋ,P}

Where ℋ is a tree-like chapter hierarchy (e.g., *Traditional Chinese Medicine Internal Medicine* → *Chapter*4: *Lung System Diseases* → *Section*   2: *Cough*), and *P* is the text content corresponding to each chapter. This structure is constructed using a document parsing model. By applying a LLM to D, we extract a set of medical logic triplets G_book_:


$${\text{G}}_{\text{book}}={\text{LLM}}_{\text{extract}}\,\left(\text{D}\right)$$

**FIGURE 3:**
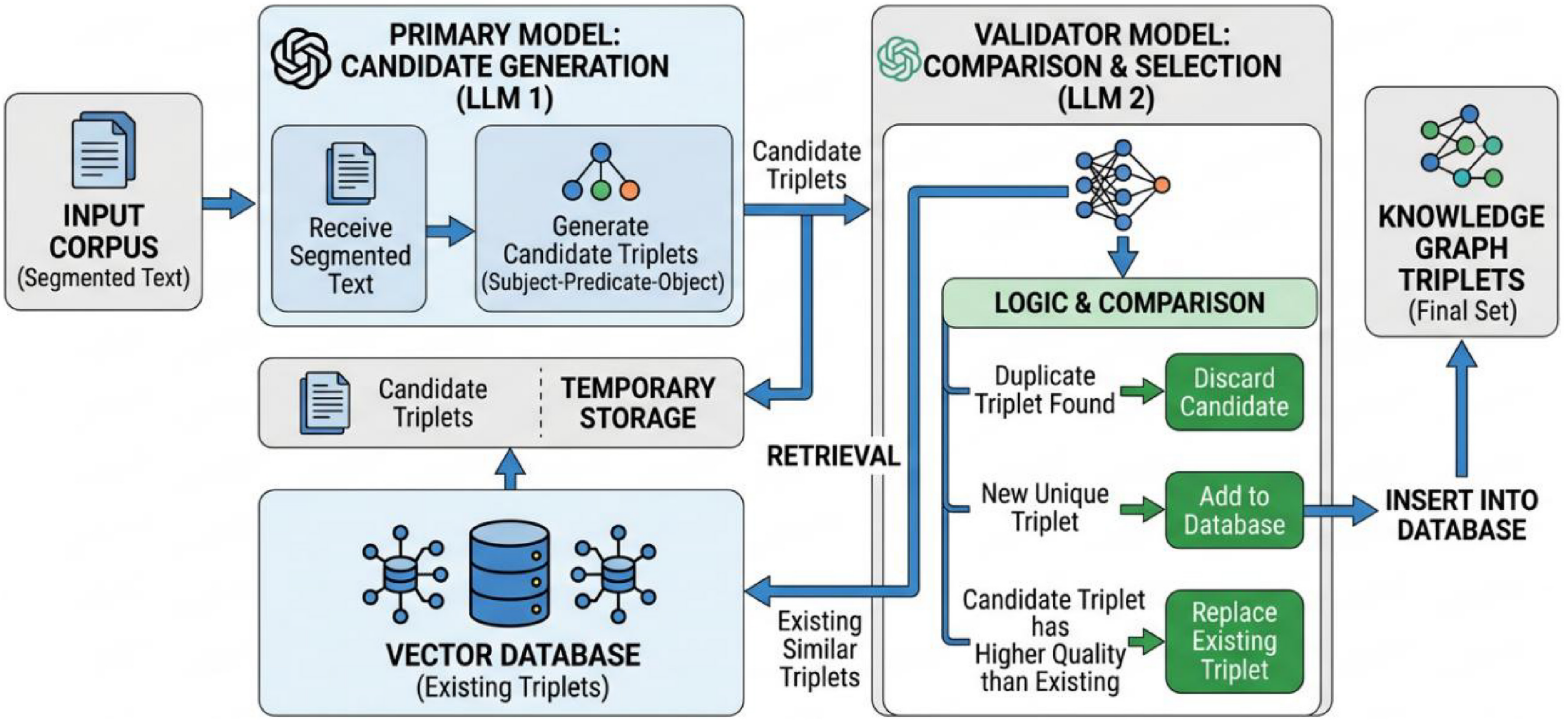
Dual-llm cooperative strategy for knowledge graph triplet extraction.

This study adopts a dual large language model collaborative strategy for knowledge graph triple extraction from segmented corpora. The specific process is as follows: qwen-plus is selected as the primary model, responsible for generating candidate triples from the input text and temporarily storing them in a vector database. deepseek-r1 is selected as the secondary model for validation, comparing and filtering the candidate triples generated by the primary model against existing triples in the database, retaining only non-redundant items for storage. If a candidate triple is of higher quality, it can replace the corresponding existing triple in the database. The specific process is shown in Figure 3.

Each triplet *t*_*k*_ ∈ *G*_book_ satisfies *t*_*k*_ = (*e*_sub_, *r*, *e*_obj_), where *e*_*sub*_ is the subject entity, *r* is the relation, and *e*_*obj*_ is the object entity. There exists a many-to-many mapping between documents and triplets, ℳ_1_ (*t*_*k*_) → *d*_1_, *d*_2_,…,*d*_*k*_, ℳ_1_ (*d*_*k*_) → *t*_1_, *t*_2_,…,*t*_*k*_, where *d*_1_, *d*_2_,…,*d*_*k*_ ∈ D.

#### 2.2.2 Enhancement and transfer of the general traditional Chinese medicine knowledge graph to a personalized knowledge graph

**FIGURE 4:**
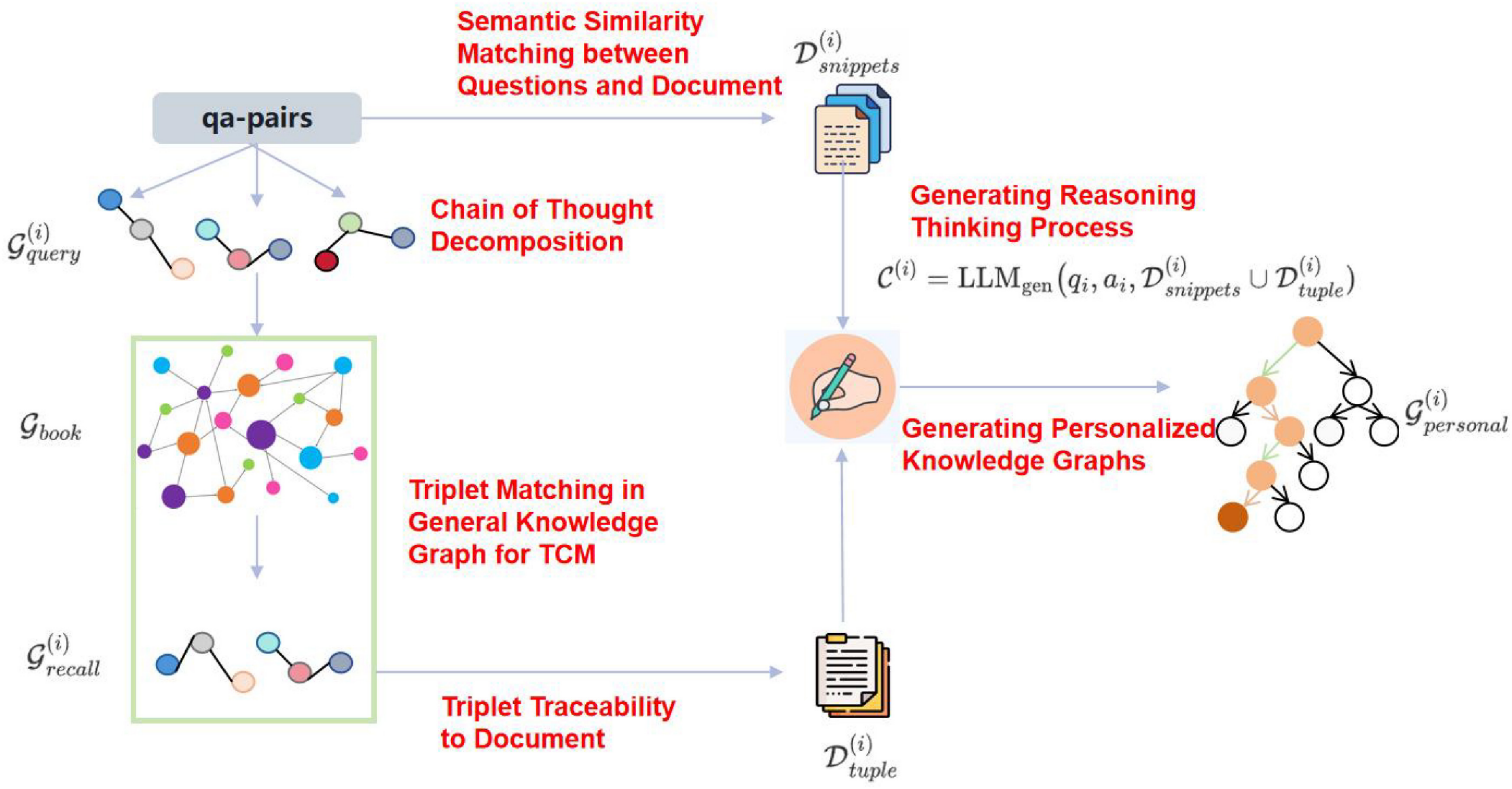
The construction process of the personalized knowledge graph.

Although we have constructed a general TCM knowledge graph through the study of TCM classics, there are significant differences in clinical diagnosis and treatment among different TCM schools and practitioners. The general TCM knowledge graph and existing RAG solutions cannot effectively capture and reflect these stylistic differences in clinical practice. Integrating diverse clinical thinking patterns into the retrieval and reasoning mechanisms of RAG remains a core challenge. Additionally, the external knowledge base required for complex clinical questions is not explicitly available and needs to be enhanced based on actual cases to improve the matching degree of the RAG knowledge base (57–62). Drawing inspiration from AgentHospital (63) and MedReason (64), we enhance and transfer the general TCM knowledge graph to obtain a personalized knowledge graph by analyzing the diagnosis and treatment cases of doctors from different schools. The specific method is shown in Figure 4:

##### 2.2.2.1 Chain of thought decomposition

Input the given question and answer into the Qwen2.5-72B-instruct model to generate a multi-hop reasoning chain and decompose it into structured triplets.

Let *Q* be the set of questions from different schools of thought, and *A*_*gold*_ be the set of their standard answers, derived from the training sets of TCM-MCQ, TCM-SD, and Jingfang-SD datasets. For *q*_*i*_ ∈ *Q* and *a*_*i*_ ∈ A_*gold*_, generate the general thinking chain triplets:


$${\text{G}}_{q\,u\,e\,r\,y}^{\left(i\right)}={\text{LLM}}_{\text{decomp}}\,\left(q_{i},a_{i}\right)$$

Where ${\text{G}}_{q\,u\,e\,r\,y}^{\left(i\right)}$ is the set of triplets decomposed from the ith question.

##### 2.2.2.2 Triplet matching and traceability

The generated triplets are aligned with the entities and mapped to the relationships in the general knowledge graph to locate the original text basis in the TCM classics. Firstly, by using vector similarity, retrieve the relevant triplets from the general knowledge graph G_book_, and obtain the recalled set ${\text{G}}_{r\,e\,c\,a}^{\left(i\right)}$:


$${\text{G}}_{r\,e\,c\,a\,l\,l}^{\left(i\right)}=\underset{t_{j}\in {\text{G}}_{\text{book}}}{\mathrm{argtop}-k}\text{sim}\,\left(\phi \,\left(t_{j}\right),\phi \,\left({\text{G}}_{q\,u\,e\,r\,y}^{\left(i\right)}\right)\right)$$

Where (⋅) is the embedding operation, and we use Alibaba Cloud’s text-embedding-v3. *sim* (⋅) is the cosine similarity. Then, we map ℳ_1_ to recall the documents in the TCM classics corresponding to the triplets:


Dt⁢u⁢p⁢l⁢e(i)=⋃tk∈Gr⁢e⁢c⁢a⁢l⁢l(i)ℳ(tk)1

##### 2.2.2.3 Questions and document

Meanwhile, for a given question *q*_*i*_, find the k most relevant text snippets from the document collection D .


$${\text{D}}_{s\,n\,i\,p\,p\,e\,t\,s}^{\left(i\right)}=\underset{d_{j}\in \text{D}}{\mathrm{argtop}-k}\text{sim}\left(\to \left(d_{j}\right),\to \left(q_{i}\right)\right)$$

##### 2.2.2.4 Generating reasoning thinking process

Use the aligned triplets and related classic texts as context to drive the Qwen2.5-72B-instruct model to generate the complete reasoning process from question to answer.


$${\text{C}}^{\left(i\right)}={\text{LLM}}_{\text{gen}}\,\left(q_{i},a_{i},{\text{D}}_{s\,n\,i\,p\,p\,e\,t\,s}^{\left(i\right)}\cup {\text{D}}_{t\,u\,p\,l\,e}^{\left(i\right)}\right)$$

##### 2.2.2.5 Generating personalized knowledge graphs

Extract new entities and relationships from the reasoning text, integrate them with the original general knowledge graph, and form a knowledge graph containing personalized clinical diagnosis and treatment features. Similar to G_book_, *C* and G_*personal*_ have a many-to-many relationship with a mapping relationship ℳ_2_ between them, where ℳ_2_ (*t*_*k*_) → {*c*_1_, *c*_2_,,*c*_*k*_} and ℳ_2_(*c*_*k*_) → {*t*_1_, *t*_2_, , *t*_*k*_}, with {*c*_1_, *c*_2_, , *c*_*k*_} ∈ C and {*t*_1_, *t*_2_, , *t*_*k*_} ∈ G_*personal*_.


$${\text{G}}_{p\,e\,r\,s\,o\,n\,a\,l}^{\left(i\right)}={\text{LLM}}_{\text{extract}}\,\left({\text{C}}^{\left(i\right)}\right)$$

**FIGURE 5:**
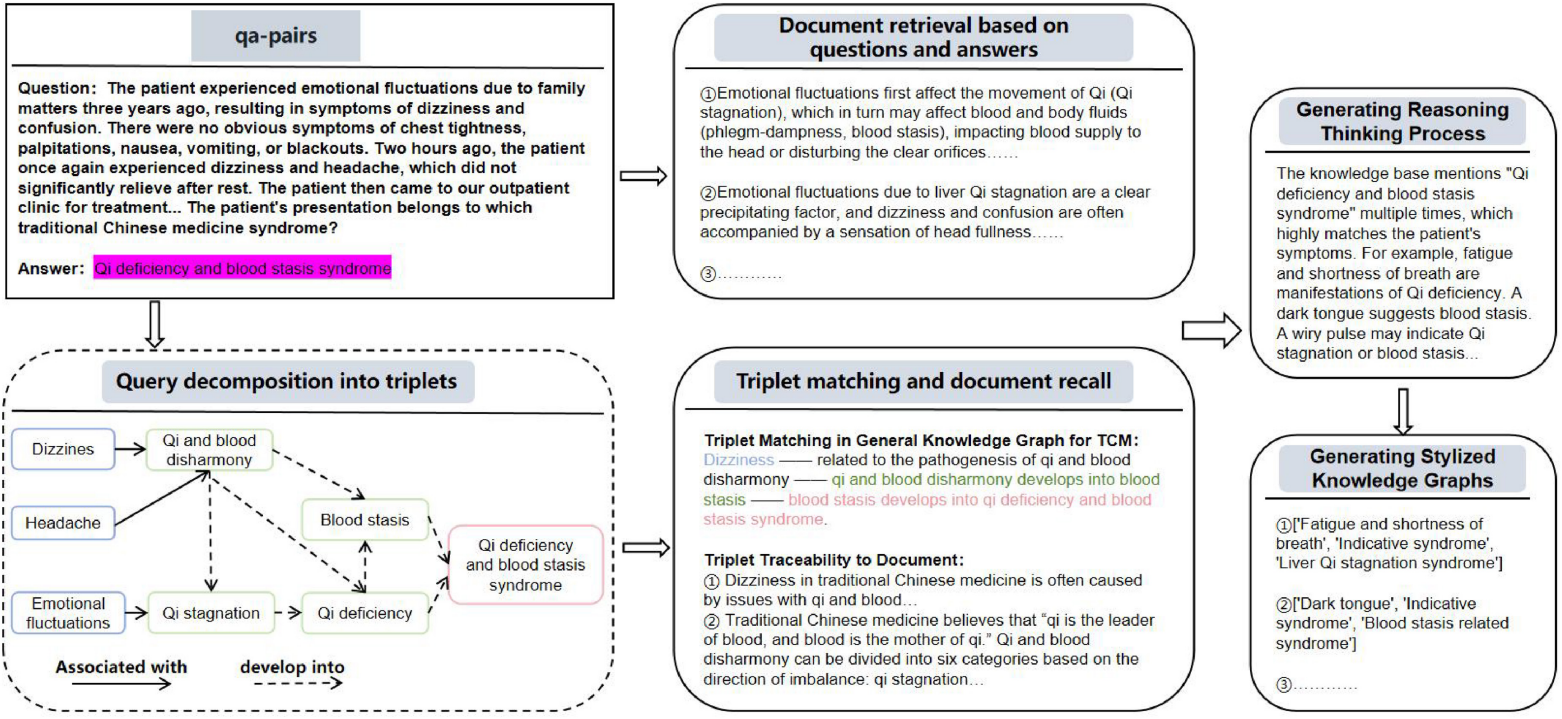
Specific examples of the construction of a personalized knowledge graph.

The advantage of constructing a personalized knowledge graph lies in the decomposition of the reasoning chain through the analysis of doctors’ actual diagnosis and treatment cases (Q&A), explicitly capturing their personalized reasoning logic (such as school preferences, emphasis on syndrome differentiation), and avoiding the homogenization defects of the general knowledge graph. At the same time, constrained by the general knowledge graph, while introducing personalized knowledge, it strictly adheres to the core authority of the traditional Chinese medicine theoretical system. More specific examples can be seen in Figure 5.

#### 2.2.3 Construction of the TCM-DiffRAG RAG architecture

After the construction of the personalized knowledge graph, we propose the TCM-DiffRAG Retrieval-Augmented Generation architecture, whose core innovation lies in: decomposing clinical questions into multi-hop triple path sequences through the chain-of-thought reasoning model, and performing semantic alignment and evidence generation based on the personalized knowledge graph. The specific steps are as follows:

##### 2.2.3.1 Chain-of-thought model training

In the previous step, for each question and answer, we generated a dataset *C* containing question-answer pairs with reasoning processes, as well as the triplets G_*style*_ obtained by decomposing the reasoning process. These two parts of the data contain a substantial amount of reasoning content, which we use to construct a supervised dataset:


DSFT={(qi,⏟ C(i))}Q⁢u⁢e⁢s⁢t⁢i⁢o⁢n-a⁢n⁢s⁢w⁢e⁢r⁢p⁢a⁢i⁢rM∪{(qi, Gp⏟⁢e⁢r⁢s⁢o⁢n⁢a⁢l(i))}C⁢h⁢a⁢i⁢n⁢o⁢f⁢t⁢h⁢o⁢u⁢g⁢h⁢t⁢t⁢r⁢i⁢p⁢l⁢e⁢t

By fine-tuning the large model parameters with domain supervision, we obtain a specialized model LLM_*cot*_ that possesses the ability to reason about traditional Chinese medicine diagnosis and treatment:


$${ℒ}_{\text{SFT}}\,\left(\theta \right)=-\underset{\left(q_{i},y_{i}\right)\in {\text{D}}_{\text{SFT}}}{\sum }\text{log}\,P_{\theta }\,\left(y_{i}|q_{i}\right)$$

We selected the Qwen2.5-7B-instruct model for fine-tuning. The specific training equipment and parameter settings are as follows: We used 8 A800 80G GPUs for training, conducted full parameter fine-tuning based on the LLaMA Factory framework, with a batch size of 2 per GPU, a learning rate of 1e-4, a warm-up ratio of 0.1, and a cutoff_len set to 2046. We used DeepSpeed ZeRO-2 to accelerate training.

##### 2.2.3.2 Multi-hop retrieval and knowledge enhancement

Chain-of-Thought Decomposition: For the input question *q*_*i*_, use LLM_*cot*_ to parse it into a multi-hop reasoning path.


$${\text{LLM}}_{\text{cot}}\left(q_{i}\right)=\left\{\left(s_{1},r_{1},o_{1}\right),,\left(s_{k},r_{k},o_{k}\right)\right\}={\text{T}}_{q\,u\,e\,r\,y}^{\left(i\right)}$$

Personalized Knowledge Recall: The triplets ${\text{T}}_{q\,u\,e\,r\,y}^{\left(i\right)}$ from multi-hop reasoning are matched with the personalized knowledge graph G_*style*_ for semantic similarity, recalling the relevant triplets $T_{r\,e\,c\,a\,l\,l}^{\left(i\right)}$.


$${\text{T}}_{r\,e\,c\,a\,l\,l}^{\left(i\right)}=\underset{t_{j}\in {\text{G}}_{s\,t\,y\,l\,e}}{\mathrm{argtop}-k}\text{sim}\,\left(\phi \,\left(t_{j}\right),\phi \,\left({\text{T}}_{q\,u\,e\,r\,y}^{\left(i\right)}\right)\right)$$

Retrieval of Provisions: ℳ_2_ realizes the mapping from triplets to reasoning text provisions C, recalling the relevant text snippets ${\text{C}}_{t\,u\,p\,l\,e}^{\left(i\right)}$.


$${\text{C}}_{t\,u\,p\,l\,e}^{\left(i\right)}=\underset{t_{k}\in {\text{T}}_{r\,e\,c\,a\,l\,l}^{\left(i\right)}}{⋃}{ℳ}_{2}\,\left(t_{k}\right)$$

##### 2.2.3.3 Traceable diagnosis and treatment decision making

For complex medical questions input by users, the large model generates enhanced responses based on the recalled personalized knowledge graph and its associated classic texts. Thanks to the deep graph traversal capability of the graph (supporting multi-hop reasoning) and the implicit associativity and scalability, the recalled content ensures both the breadth of structured knowledge and the depth of traceable reasoning, thereby integrating the dual advantages of both knowledge graph-based RAG and reasoning-based RAG.


$${\overbrace{y}}_{i}={\mathrm{LLM}}_{\mathrm{gen}}\left(q_{i},\underset{Reasoning\;path}{\underbrace{T_{recall}^{\left(i\right)}}}\;,\underset{Text\;source}{\underbrace{C_{tuple}^{\left(i\right)}}}\right)$$

## 3 Results

### 3.1 Dataset and knowledge graph construction

We divide the corpus dataset into four types: TCM books, TCM-MCQ, TCM-SD, and Jingfang-SD, as shown in Table 1.

**TABLE 1:** Composition of the corpus database.

Corpus	Snippets	Average tokens	Average triples
TCM books	433,950	330	8
TCM-MCQ_training set	21,660	103	12
TCM-MCQ_test set	600	117	/
TCM-SD_training set	43,085	409	16
TCM-SD_test set	5,486	416	/
Jingfang-SD_training set	20,049	194	16
Jingfang-SD_test set	5,012	194	/

The TCM Books Corpus constitutes the foundational General TCM Knowledge Graph. This corpus includes 580 TCM works, which are divided into macro and micro knowledge graphs through specific methods. The text snippets in the corpus (totaling 433,950) correspond to different hierarchical titles in the books, forming the nodes of the macro knowledge graph. Each text snippet (with an average length of about 330 tokens) represents the specific text content under a certain hierarchical title. We use large language models to extract triple knowledge from each text snippet, with an average of 8 triples extracted per snippet.The remaining three corpora (TCM-MCQ, TCM-SD, Jingfang-SD) represent different difficulty levels for Retrieval-Augmented Generation (RAG) task evaluation benchmarks:

- TCM-MCQ Corpus: Focuses on testing the mastery of general TCM knowledge. This dataset is derived from TCM medical examination question books, and the task requires selecting the only correct answer from five options. Most of the answer information can be directly retrieved from the TCM books corpus, making this dataset represent the lowest task difficulty.
- TCM-SD Corpus: This open-source dataset originates from the real medical records of Xuzhou Hospital of TCM (26). The task is to determine the only correct answer from 148 candidate syndromes. Compared to TCM-MCQ, the RAG difficulty of this dataset is significantly increased, as answers are typically not directly obtainable from the books corpus and require reasoning based on the recalled book snippets.
- Jingfang-SD Corpus: This private dataset comes from the outpatient cases of the Second Affiliated Hospital and the Third Affiliated Hospital of Zhejiang Chinese Medical University. The task requires selecting the only correct answer from 42 candidate syndromes. Its syndrome differentiation approach differs from TCM-SD (based on general principles like the Eight Principles, Zang-Fu organs, meridians, Qi, blood, body fluids, or Sanjiao) and is rooted in the TCM Classical Formula school, with distinct school characteristics. Due to the relative scarcity of literature recording such characteristic syndrome differentiation experiences, only a small number of relevant text snippets are available for recall in the books corpus, posing the greatest challenge to the RAG system.

The above three evaluation benchmarks (TCM-MCQ, TCM-SD, Jingfang-SD) are divided into training sets and test sets. The training sets are used to construct the personalized knowledge graph, while the test sets are used to evaluate the effectiveness of the RAG system. The Jingfang-SD dataset used in this study was constructed independently. To strictly avoid data leakage between the training and test sets, we adopted a temporal partitioning strategy to build the dataset: outpatient data from January 2024 to December 2024 were included as the training set, and patient data from January 2025 to April 2025 were used as the test set. Furthermore, we deduplicated patient IDs, retaining only the first-visit record for each patient, resulting in 20,049 training samples and 5,012 test samples.

### 3.2 Evaluation of the general traditional Chinese medicine knowledge graph effectiveness

The quality of the corpus, preprocessing, and the method of graph construction have a significant impact on RAG performance. Given that the general Traditional Chinese Medicine (TCM) knowledge graph is the foundational premise for constructing the personalized knowledge graph, it is crucial to evaluate the advancement of this general knowledge graph construction method through experiments. For this purpose, we chose RAGAS (65) as the evaluation framework. This is a tool designed to automate the evaluation of RAG system effectiveness. As shown in Table 2, this study uses OpenAI’s gpt-3.5-turbo-16k as the base large language model (LLM), the test set is selected from the TCM-MCQ test set, Alibaba Cloud’s text-embedding-v3 is used as the embedding model, and the number of recalled documents is set to k = 20. A systematic evaluation was conducted on different corpus processing methods. The experiment compared the following four methods:

**TABLE 2:** RAGAS evaluation of the general traditional Chinese medicine knowledge graph on the TCM-MCQ test set.

Knowledge graph construction methods	Accuracy	Answer similarity	Context precision	Context recall	Context entity recall
Without RAG	0.403	0.786	\	\	\
Fixed character segmentation	0.540	0.856	0.621	0.829	0.173
Macro knowledge graph segmentation	0.640	0.863	0.808	0.848	0.188
Micro knowledge graph segmentation	0.627	0.871	0.782	0.836	0.192
Macro-micro knowledge graph integrated retrieval	0.687	0.885	0.846	0.887	0.244

- Without RAG: Relying solely on the LLM’s own knowledge to generate predicted answers (without retrieval enhancement).
- Fixed Character Segmentation: The TCM books corpus text is segmented into fixed lengths (approximately 330 tokens), resulting in 435,316 document segments. The input questions and document segments are recalled based on semantic similarity after embedding processing.
- Macro Knowledge Graph Segmentation: Segmentation is performed based on the original title hierarchy of the books, generating 433,950 document segments. This method typically produces segments that include titles and their corresponding text content, offering better semantic completeness. Document retrieval is calculated based on cosine similarity: ${\text{D}}_{\text{snippets}}^{\left(i\right)}={\mathrm{argtop}-k}_{d_{j}\in \text{D}}\text{sim}\,\left(\phi \,\left(d_{j}\right),\phi \,\left(q_{i}\right)\right)$, where ϕ represents the embedding function.
- Micro Knowledge Graph Segmentation:
  1. First, perform semantic matching retrieval of questions in the triplet set of the general TCM knowledge graph G_book_: ${\text{G}}_{\text{recall}}^{\left(i\right)}={\mathrm{argtop}-k}_{t_{j}\in {\text{G}}_{\text{book}}}s\,i\,m\,\left(\phi \,\left(t_{j}\right),\phi \,\left(q_{i}\right)\right)$.
  2. Subsequently, retrieve the original text snippets corresponding to the matched triplets based on a predefined triplet-text snippet mapping ℳ_1_: ${\text{D}}_{\text{tuple}}^{\left(i\right)}={⋃}_{t_{k}\in {\text{G}}_{\text{recall}}^{\left(i\right)}}{ℳ}_{1}\,\left(t_{k}\right)$.
- Macro-Micro Knowledge Graph Integrated Retrieval: Combine the macro-level snippet retrieval ${\text{D}}_{s\,n\,i\,p\,p\,e\,t\,s}^{\left(i\right)}$ from method (3) with the micro-level triplet-associated snippet retrieval ${\text{D}}_{\text{tuple}}^{\left(i\right)}$ from method (4), and take their union as the final retrieved document set: ${\text{D}}_{\text{final}}^{\left(i\right)}={\text{D}}_{\text{snippets}}^{\left(i\right)}\cup {\text{D}}_{\text{tuple}}^{\left(i\right)}$.

The experimental results show that the macro-micro knowledge graph integration method leads in all evaluation indicators, verifying the significant advancement of the knowledge graph method we constructed.

Currently, fixed character segmentation is still the method used by most research. To ensure the comparability of document length with subsequent knowledge graph segmentation methods, this study uniformly sets the segmentation at approximately 330 characters. Although this method is simple to implement, it easily leads to semantic fragmentation and entity disconnection. A typical problem is that a large number of tables in textbooks carry key information, and related questions in the test set require complete tables to obtain answers. Fixed character segmentation often truncates tables, destroying their structural integrity and semantic coherence.

In contrast, macro knowledge graph segmentation relies on the original hierarchical structure of books, effectively ensuring the integrity of semantic units. This method performs better when dealing with questions that require cross-document comparison (e.g., what is the preferred treatment plan for low back pain caused by dampness and heat?). However, its retrieval depth has limitations, and its effectiveness may be reduced when the question involves knowledge points buried in text details (e.g., in the Guizhi Decoction and its modified formulas, how does the proportion of Guizhi and Shaoyao change according to clinical conditions?) or when the hierarchical structure causes key entity information to be ineffective.

The advantage of micro knowledge graph segmentation is its ability to relatively accurately match entity relationships. However, it should be noted that the current method directly matches the similarity between questions and triples using vector similarity, lacking an explicit step for extracting key entities in the question, which results in its overall performance being slightly inferior to that of the macro knowledge graph segmentation, showing some improvement only in the Context Entity Recall indicator.

The macro-micro knowledge graph integration method combines the advantages of both: the macro level retains the logical framework and semantic integrity of syndrome differentiation and treatment, while the micro triples precisely lock the core knowledge entities. This synergistic effect enables it to achieve the best comprehensive performance among the four RAG methods.

### 3.3 Evaluation of the thinking chain model’s effectiveness

**FIGURE 6:**
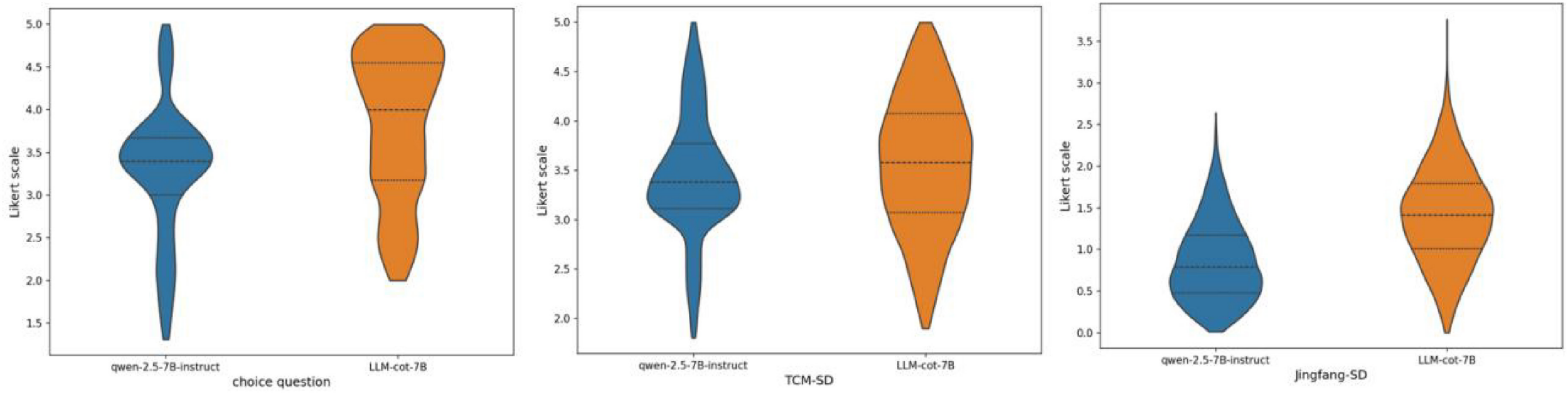
Comparison of the quality of triplet decomposition by different thinking chain models.

Using the datasets *C* and G_*personal*_, which contain the thinking process behind questions, we fine-tuned the qwen-2.5-7B-instruct model to obtain LLM-cot-7B. To evaluate LLM-cot-7B’s ability to decompose questions, we selected deepseek-r1 as the reference model to assess the quality of the question-related triples generated by qwen-2.5-7B-instruct and LLM-cot-7B. We used the Likert Scale as the evaluation metric, scoring the triples generated by the two models on a scale from 0 to 5. As shown in Figure 6, we conducted an independent samples *t*-test for statistical significance analysis, which revealed that the quality of triples generated by the fine-tuned LLM-cot-7B was significantly superior to that of qwen-2.5-7B-instruct (*P* < 0.05).

In addition to having better question decomposition capabilities, LLM-cot-7B also has the ability to directly answer questions. The results on the TCM-SD test set show that the effectiveness of LLM-cot-7B (0.74) is significantly better than that of previous studies (0.52) (13). This confirms that fine-tuning LLMs with data on the thinking process, in addition to the final answer data, can achieve better results. Further ablation experiments indicate that TCM-DiffRAG, using LLM-cot-7B as the thinking chain model in combination with a personalized knowledge graph, outperforms the use of LLM-cot-7B alone in all three test sets.

### 3.4 Evaluation of TCM-DiffRAG’s ablation experiment

**FIGURE 7:**
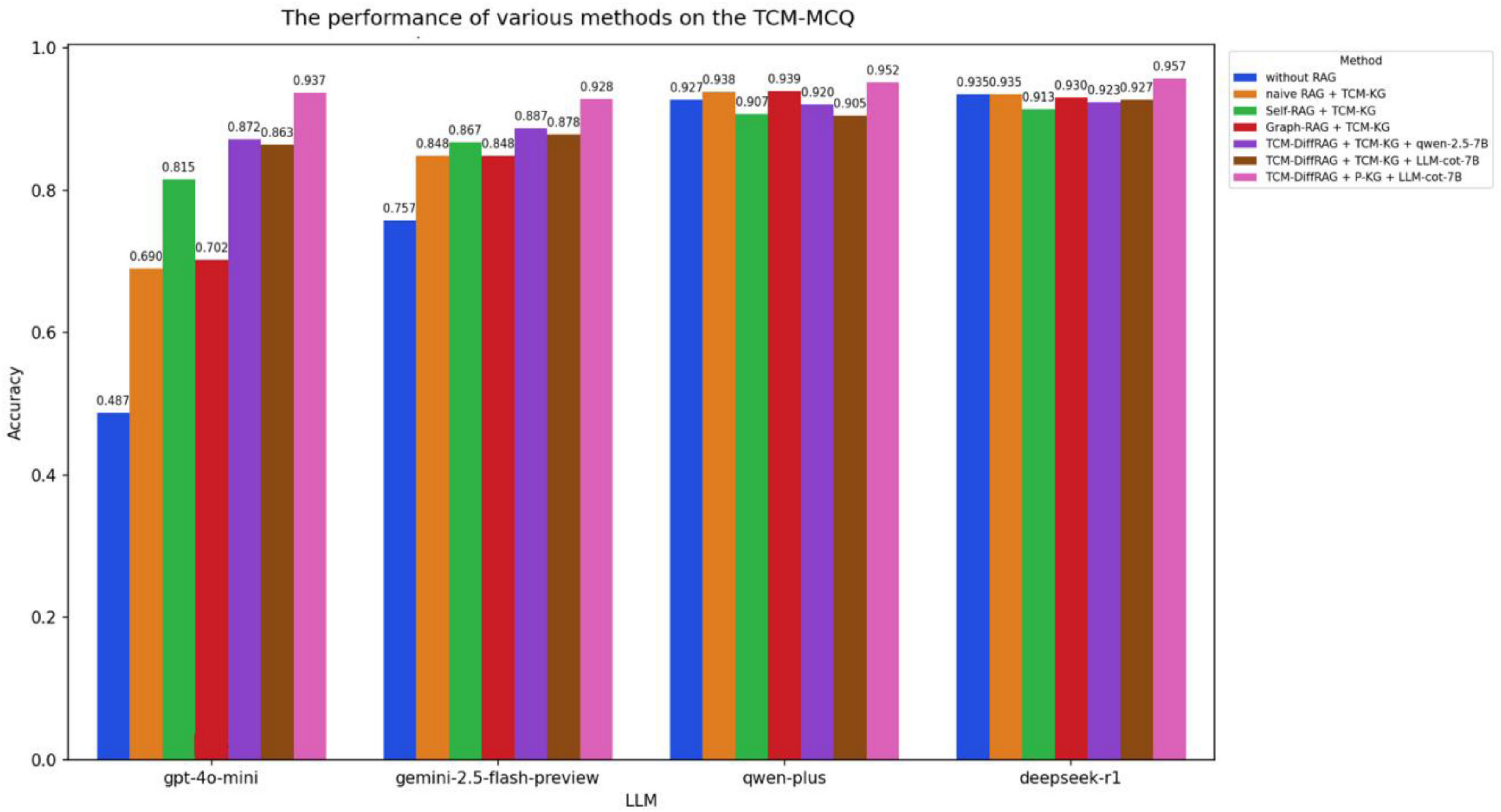
Performance of different RAG methods on the TCM-MCQ test set.

**FIGURE 8:**
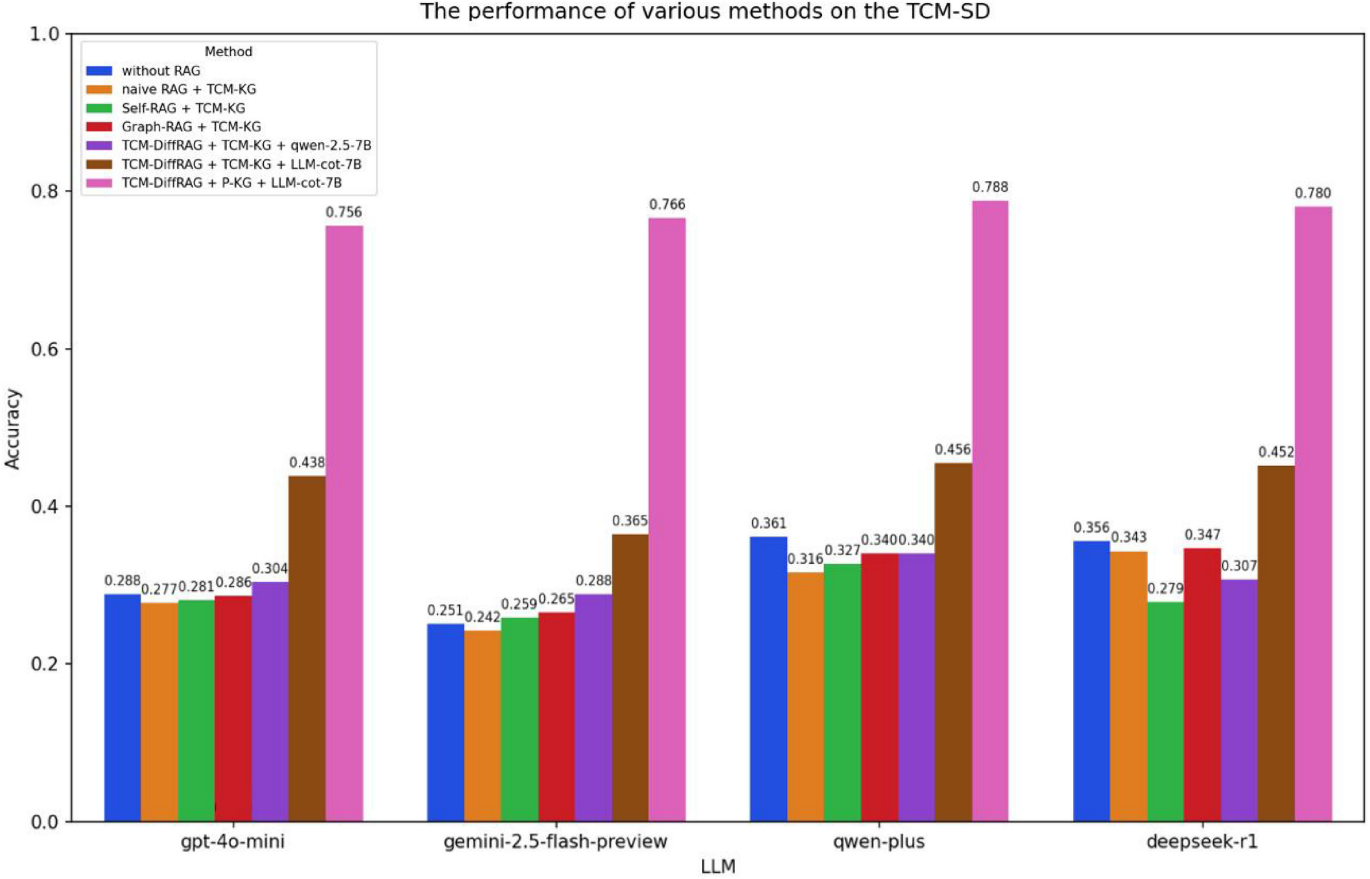
Performance of different RAG methods on the TCM-SD test set.

**FIGURE 9:**
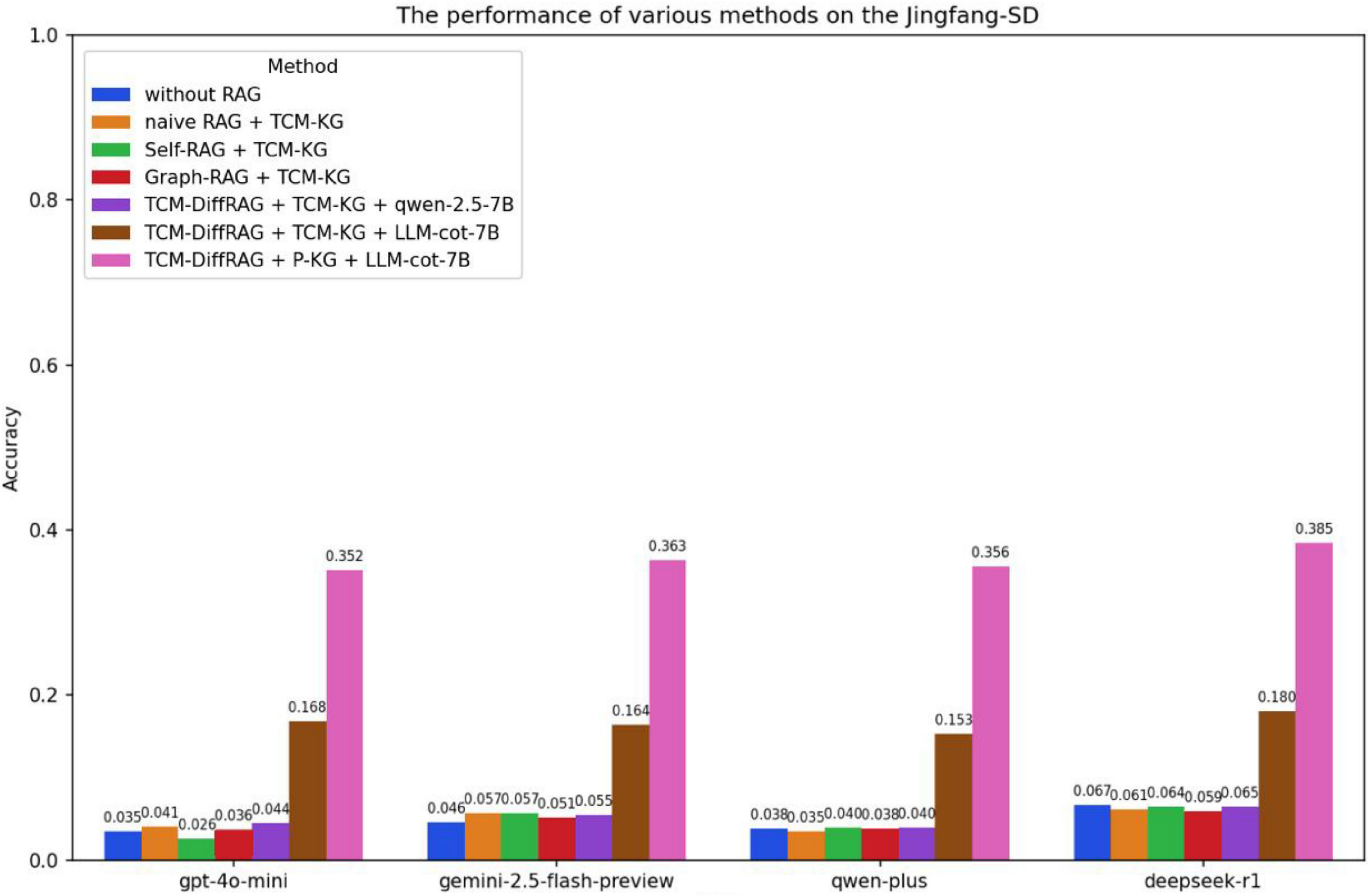
Performance of different RAG methods on the Jingfang-SD test set.

We employed the McNemar test to assess the statistical significance of the differences between TCM-DiffRAG and the baseline model. As seen in Figures 7–9, when relying solely on the models’ own capabilities, qwen-plus and deepseek-r1, which are primarily trained on Chinese language datasets, significantly outperform gpt-4o-mini and gemini-2.5-flash-preview on the TCM-MCQ and TCM-SD tasks. However, on the Jingfangjing-SD test set, all four LLMs perform poorly, indicating that none of them have learned this type of personalized syndrome differentiation thinking during training. Based on the performance of the large models alone, the three test sets represent three different levels of difficulty. The performance of the LLMs is significantly improved when the TCM-DiffRAG method, based on Personalized-KG and LLM-cot-7B is added (*p* < 0.01).

In the TCM-MCQ test set (Figure 7), both gpt-4o-mini and gemini-2.5-flash-preview achieve significant improvements with different RAG methods, while qwen-plus and deepseek-r1 only see improvements when using the TCM-DiffRAG method with LLM-cot-7B and a personalized knowledge graph. This may be because qwen-plus and deepseek-r1 already possess excellent general TCM knowledge capabilities, and ordinary RAG methods introduce noisy recalls, leading to negative effects. Only by adding LLM-cot-7B as the thinking chain model and using a more specialized personalized knowledge graph as the knowledge base can these models achieve certain improvements.

The difficulty of the TCM-SD dataset is higher (Figure 8), and ordinary RAG methods are not effective in improving the performance of LLMs. Significant improvements are observed only after applying the TCM-DiffRAG method. When using LLM-cot-7B as the thinking chain generation model, there is a notable performance improvement compared to the original model. We believe this is because TCM-SD, as a clinical practice test set, requires a high level of reasoning ability from the model. LLM-cot-7B can decompose input queries into finer-grained triples and reason through them. These interconnected triples form a knowledge graph structure with a clinical thinking chain. This method balances the breadth of information retrieval of RAG with the deep reasoning capabilities of the thinking chain.

Jingfang-SD is the most challenging among the three test sets (Figure 9). Without using RAG, the four generation models can only achieve an accuracy of 0.03–0.07, and there is no significant improvement even with ordinary RAG methods. However, when using LLM-cot-7B in combination with a personalized knowledge graph, the accuracy is increased to 0.35–0.38. The possible reasons for this, in addition to the LLMs’ lack of reasoning ability in classical formula diagnosis and treatment, may be the general knowledge graph’s lack of classical formula diagnosis and treatment data. More significant improvements can be achieved after using a personalized knowledge graph.

## 4 Discussion

### 4.1 Innovativeness

Firstly, the personalized Traditional Chinese Medicine (TCM) diagnosis and treatment scenario we focus on has been overlooked in previous research related to RAG. Prior work has mostly enhanced RAG performance by incorporating knowledge graphs as knowledge bases or improving reasoning-enhanced retrieval capabilities. However, in the specific context of TCM clinical diagnosis and treatment, the following issues persist. The primary challenge is the contradiction between general knowledge and the construction of personalized knowledge. Most existing studies have focused on building general TCM knowledge graphs, failing to fully account for the personalized variations among doctors during the diagnostic process. Since the performance of RAG highly depends on the quality of the knowledge base, general TCM knowledge graphs struggle to adapt to the practical needs of different doctors. Therefore, based on a general TCM knowledge graph, this study constructs targeted personalized knowledge graphs by integrating the historical case data of different doctors. This method not only ensures the traceability of personalized knowledge but also reflects the diagnostic differences among doctors. In practical application, the RAG system can select the corresponding personalized knowledge graph based on the current doctor, thereby significantly improving retrieval accuracy and adaptability. This holds significant practical value in the TCM context, which emphasizes personalized treatment.

Secondly, our data processing method for the corpus text differs. In existing Graph RAG research, most methods perform semantic segmentation on the corpus using fixed-length characters or paragraphs, and then extract knowledge graph triples from the segmented text. For example, ZhiFangDanTai (66) sets the text chunk size to 512 tokens; TCM MLKG-RAG (42), MedGraphRAG (39), and OpenTCM (67) segment based on paragraphs. In contrast, our method segments documents according to the hierarchical chapter structure of the books, using units like “book-volume-chapter-section” as nodes. Compared to fixed-character or paragraph-based segmentation, our approach preserves the semantic coherence of the original text, enabling better establishment of associations between distant text fragments and across heterogeneous corpora. For instance, different books may discuss the same disease; our method can better capture such connections.

Thirdly, the framework for constructing the knowledge graph is different. OpenTCM (67) and ZhiFangDanTai (66) utilize open-source Graph RAG frameworks. Although Graph RAG can perform functions like community classification and community summarization on the extracted knowledge graph, relying entirely on LLMs for corpus summarization is akin to a black box and carries uncertainty. Our framework for extracting the knowledge graph is built from scratch. The chapter-level hierarchy serves as the macro knowledge graph, a concept similar to community classification and summarization, but it offers greater determinism and reliability compared to the Graph RAG framework. On this macro knowledge graph foundation, we further perform fine-grained triple extraction on the text paragraphs corresponding to each node, constructing a micro knowledge graph centered on entities and relationships. The macro graph represents the overall knowledge framework and contextual logic of the medical classics, while the micro graph depicts specific medical concepts and relationships. The organic integration of the two constitutes a general TCM knowledge graph that covers multi-granularity knowledge.

Finally, existing reasoning-based RAG methods often rely on prompt engineering, guiding LLMs to decompose complex questions into sub-questions for stepwise retrieval and reflection. For example, Self-BioRAG (48) possesses reflection capabilities, judging whether a question requires further retrieval, whether retrieved passages are relevant, and whether the generated content is reasonable. i-MedRAG (47) allows the Large Language Model (LLM) to iteratively generate follow-up queries based on previous retrieval results, gradually deepening information exploration to form a “retrieval history,” which is ultimately used to generate the final answer. HiRMed (68) also employs a tree structure, performing medical reasoning at each node during the QA process. However, crafting dedicated prompts for each doctor is neither feasible nor scalable, and the multi-step reasoning process significantly increases time overhead. To address this, we propose training a CoT model based on personalized knowledge graphs. This model can adapt to the aforementioned three test scenarios, automatically decomposing the input question into a multi-hop query triple sequence that aligns with a specific diagnostic style, and subsequently retrieving the relevant subgraph structures, thereby achieving efficient and personalized knowledge retrieval and reasoning.

### 4.2 Qualitative analysis

As shown in Table 3, we take one data entry from TCM-SD as an example. Chief Complaint: Epigastric pain for over a year, with recurrence for 1 month. History of Present Illness: One year ago, the patient experienced persistent dull pain in the epigastric region without obvious cause, which relieved after eating. There was no referred pain to the shoulder or back, but occasional epigastric burning sensation was reported. The patient denied acid reflux or belching. Abdominal bloating occurred occasionally, with no nausea, vomiting, or fever. Appetite was poor, but sleep was fair. Urination was normal, and bowel movements occurred once daily without mucus or blood in the stool. The patient sought treatment at a local hospital and improved after oral medication (specific drugs unknown). Over the past year, the epigastric pain recurred repeatedly, and symptoms resolved each time with oral medication. One month ago, the pain recurred. After oral treatment at a local hospital, symptoms did not improve significantly. The patient presented to our hospital for further diagnosis and treatment and was admitted to our department via the outpatient clinic. Upon admission: Epigastric pain, described as intermittent dull pain. No referred pain to the shoulder or back, no acid reflux, belching, or retrosternal burning sensation. Abdominal bloating occurred occasionally. No nausea, vomiting, fever, dizziness, palpitations, or chest tightness. Appetite was poor, sleep was good, urination was normal, and bowel movements occurred once daily without mucus or blood in the stool. There was no significant recent weight change. Tongue: red with yellow coating. Pulse: wiry. Syndrome Diagnosis: Liver-Stomach Stagnation-Heat Syndrome (肝胃郁热证).

**TABLE 3:** Comparison of different personalized KG and reasoning approaches for the same case.

Personalized KG	Prediction	Reasoned triples	Retrieved personalized KG
P-KG base on TCM-MCQ_training Set	肝气犯胃证	[(“肝郁气滞,” “表现为,” “上腹疼痛”), (“肝郁气滞,” “表现为,” “脉弦”),…… (“肝气犯胃,” “由,” “肝郁气滞导致”), (“肝气犯胃,” “表现为,” “上腹疼痛进食后缓解”)]	[(“肝郁气滞,” “主要临床表现为,” “胸胁、少腹胀痛”), (“肝郁气滞,” “表现为,” “脉弦”), ……(“肝气犯胃,” “核心病机为,” “肝气横逆犯胃，胃失和降”), (“肝气犯胃,” “主要临床表现为,” “脘胁胀闷疼痛”)]
P-KG base on TCM-SD_training set	肝胃郁热证	[(“无明显两胁胀闷,” “排除证候,” “肝气郁结证”), (“无胃脘灼痛，无喜冷饮，无发热口渴，无便秘,” “排除证候,” “脾胃积热证”), …… (“肝胃郁热证与脾胃积热证,” “相同点,” “均有胃部不适表现”), (“肝胃郁热证与脾胃积热证,” “鉴别点,” “后者有明显热盛症状如喜冷、便秘”),……]	[(“无明显胸闷,” “排除证候,” “肝郁气滞证”), (“无发热口渴、便秘,” “排除证候,” “脾胃积热证”),……(“肝胃郁热证与脾胃积热证,” “区别点,” “前者以郁热为主，后者以脾胃实热为主”)……]
P-KG base on Jingfang-SD_training set	少阳阳明合病	[(“上腹疼痛，进食后缓解,” “提示病机,” “胃虚”)， (“上腹部烧灼感、舌红苔黄,” “提示病机,” “里热”)， (“脉弦、腹胀偶作,” “提示病机,” “气滞”)， (“食纳欠佳,” “提示病机,” “胃气不和”)， (“里热+胃虚+气滞,” “提示证候,” “少阳阳明合病”)……]	[(“胃虚,” “临床表现,” “胃脘隐痛或不适”)， (“里热,” “临床表现,” “舌质红苔黄”)， (“气滞,” “临床表现,” “腹胀”)， (“胃气不和,” “临床表现,” “食欲差”)， (“少阳阳明合病,” “包含,” “里热、胃虚、气滞”)......]

We inferred the syndrome for this case based on different personalized knowledge graphs and reasoning models. After inputting the question, a CoT model generated triples. The CoT model was trained via the “2.2.3.1 Chain-of-Thought Model Training” step, and training on different datasets yielded triples with different styles. To mitigate hallucinations, the next step involved retrieving the reasoned triples from the personalized knowledge graph via similarity matching. Finally, large language models such as qwen-plus and deepseek-r1 generated responses based on the retrieved content. In the first test case, we performed inference using the personalized knowledge graph derived from the TCM-MCQ_Training Set, resulting in the predicted syndrome “Liver Qi Attacking the Stomach (肝气犯胃证).” The inference triples were generated by the CoT model trained on data fused from TCM-MCQ_Training Set and TCM-KG. Since the reasoning logic and the personalized knowledge graph were not fully compatible with TCM-SD, the prediction failed. In the second case, the model performed inference using the CoT model trained on the TCM-SD_Training Set. Compared to the first method, the reasoned triples now included analysis of negative symptoms and syndrome differential diagnosis, ultimately guiding the model to the correct answer: “Liver-Stomach Stagnation-Heat Syndrome (肝胃郁热证).” The third case employed the reasoning style and personalized knowledge graph from Jingfang-SD. The reasoning style changed significantly, focusing more on the connections between symptoms and pathogenesis. The syndrome diagnosis shifted from Eight-Principle Pattern Differentiation to Six-Channel Pattern Differentiation.

### 4.3 Limitations

The fine-tuned CoT model deployed in this study has a scale of 7B parameters. Without quantization, it requires approximately 28GB of GPU memory. After 8-bit quantization, this is reduced to 8GB, and further decreased to 4–6GB with 4-bit quantization (69). The reduction in memory demand enables the model to be deployed on ordinary personal computers, demonstrating its potential feasibility for practical application in future clinical environments. Simultaneously, this study has several limitations: 1. During the knowledge graph triple extraction process, no explicit schema constraints were defined. This approach benefits the large language model in fully mining fine-grained textual information, enhancing the comprehensiveness of subsequent retrieval, thus improving the utility of the knowledge graph, but it sacrifices the knowledge graph’s rigor. 2. The study only evaluated the knowledge graph from the perspective of its utility as a knowledge base, lacking a systematic assessment of intrinsic metrics of the knowledge graph (such as relationship accuracy, entity correctness, etc.). 3. Experiments were only validated on two TCM datasets with different styles, TCM-SD and Jingfang-SD. Whether this method can be generalized to more differentiated TCM datasets requires further investigation. 4. The fine-tuned Chain-of-Thought model is based solely on a 7B-scale base model, with no ablation experiments or comparisons conducted on large language models of different scales and architectures. 5.We solely employed LLMs to evaluate the reasoning quality of the Cot model, lacking expert evaluation. 6. The effectiveness of this method in real clinical scenarios still needs to be verified by subsequent clinical studies. The aforementioned aspects represent important directions for future improvements.

## Data Availability

The data analyzed in this study is subject to the following licenses/restrictions: Part of the code, prompts, and data are provided in Supplementary Appendices 1–5 and can be accessed for reproducibility on GitHub at: https://github.com/LiJianmin6706/Tcm_Diff_RAG/tree/main. Requests to access these datasets should be directed to Shuyuan Lin, lin_shuyuan@foxmail.com.
