# Supplementary material for: TCM-DiffRAG: personalized syndrome differentiation reasoning method for traditional Chinese medicine based on knowledge graph and chain of thought
Source: Front Med (Lausanne). 2026 Apr 21;13:1804478. doi: 10.3389/fmed.2026.1804478 (PMC13140767; doi:10.3389/fmed.2026.1804478)
Supplement: Supplementary file 1 [file Data_Sheet_1.zip › Appendix/Appendix3/demo.html]

检索子图
