# Supplementary material for: TCM-DiffRAG: personalized syndrome differentiation reasoning method for traditional Chinese medicine based on knowledge graph and chain of thought
Source: Front Med (Lausanne). 2026 Apr 21;13:1804478. doi: 10.3389/fmed.2026.1804478 (PMC13140767; doi:10.3389/fmed.2026.1804478)
Supplement: Supplementary file 1 [file Data_Sheet_1.zip › Appendix/Introduction to the Appendix 1 to 5.docx]

**Introduction to the Appendix 1 to 5**

1. **Appendix 1 and Appendix 2**

Appendix 1 and Appendix 2 contain the code for constructing the general Traditional Chinese Medicine (TCM) knowledge graph. Specifically, Appendix 1 pertains to performing OCR text recognition and constructing chapter hierarchies for books, while Appendix 2 includes the prompts used for triple extraction from the corpus. The workflow of Part 1 is as follows: PDF files are converted to images; different page elements (such as titles, text paragraphs, images, etc.) are identified via object detection; text is recognized using the PaddleOCR-VL model; the document titles are hierarchically processed by a large language model; and finally, a Markdown document is output. The input PDF books are ultimately processed into Markdown documents with a clear hierarchical title structure. Subsequently, the documents are segmented into multiple text paragraphs based on these title levels, and triples are extracted using a large language model. The code for these two parts corresponds to the content described in "2.2.1. Construction of the General TCM Knowledge Graph".

1. **Appendix 3**

Appendix 3 details the retrieval process. Initially, it is necessary to configure the Chain-of-Thought (CoT) model, the embedding model, and the Milvus vector database. The workflow is as follows: an input question is decomposed into triples by the CoT model; these triples are then used for retrieval within the personalized knowledge graph, while the original input question is simultaneously used for retrieval in the general knowledge graph; the source text passages of the retrieved triples are traced back; and finally, the ultimate recall is obtained. This process corresponds to the content in "2.2.2. Enhancement and Transfer of the General Traditional Chinese Medicine Knowledge Graph to a Personalized Knowledge Graph".

In demo.html, I display a schematic diagram of the retrieval process. In the diagram, the red dot represents the input question. The Chain-of-Thought (CoT) model decomposes the question into triples. The green nodes represent the successfully matched content from the personalized knowledge graph, while the blue nodes represent the successfully matched content from the general knowledge graph. Hovering the mouse over a blue node displays the specific source text for the retrieved information.

1. **Appendix 4**

Appendix 4 contains the code for constructing the CoT training templates. Jingfang_train.xlsx is an example of the dataset. We utilize the data from the chief_complaintand symptomcolumns to generate questions for syndrome determination. The diacolumn provides the syndrome answers, and the tuplecolumn contains the Chain-of-Thought (CoT) data, which is derived by retrieving information based on the questions and answers. We use these three parts of the data to construct the SFT (Supervised Fine-Tuning) dataset.

1. **Appendix 5**

Appendix 5 contains the code for the McNemar test comparing TCM-DiffRAG with the baseline model.
